# Analysis of peripheral inflammatory T cell subsets and their effector function in patients with Birdshot Retinochoroiditis

**DOI:** 10.1038/s41598-021-88013-0

**Published:** 2021-04-21

**Authors:** Janine Trombke, Lucie Loyal, Julian Braun, Uwe Pleyer, Andreas Thiel, Dominika Pohlmann

**Affiliations:** 1 Regenerative Immunology and Aging, BIH Center for Regenerative Therapies, Charité Universitätsmedizin Berlin, Berlin, Germany; 2 Charité-Universitätsmedizin Berlin, Corporate Member of Freie Universität Berlin and Humboldt-Universität Zu Berlin, Augustenburger Platz 1, 13353 Berlin, Germany; 3 Si-M/“Der Simulierte Mensch” a Science Framework of Technische Universität Berlin and Charité-Universitätsmedizin Berlin, Berlin, Germany; 4 Berlin Institute of Health at Charité-Universitätsmedizin Berlin, Charitéplatz 1, 10117 Berlin, Germany

**Keywords:** Chronic inflammation, Chemokines, Autoimmunity

## Abstract

Birdshot Retinochoroiditis (BSRC) is a progressive non-infectious intraocular inflammation that affects choroid and retina. Inflammatory processes have adverse effects on vision by affecting photoreceptor-bearing cells that do not regenerate. This study aimed at characterizing inflammatory CD4^+^ and CD8^+^ T cell subsets in the peripheral blood of active and inactive BSRCs. Furthermore, we correlated phenotypical and functional immunological analyses with clinical data. We observed a slight increase of terminally differentiated effector memory CD8^+^ T cells expressing CD45RA (T_EMRA_) in blood of inactive, compared to active BSRCs. Moreover, we identified a trend for a decreased population of T_H_2 cells and increased T_H_1 frequencies in active BSRCs, a typical sign of ongoing autoimmune processes. Functional assays demonstrated severe and overall impairment of effector function of both, CD4^+^ and CD8^+^ inflammatory T cells, which might reflect T cell exhaustion. Although the eye is the main site of inflammation in BSRC, we observed altered T cell subset compositions in the peripheral blood, dependent on the disease status. Our results indicate that T cells may play a major role in BSRC pathology, although our cohort size is too limited for definitve conclusions. Future studies with larger BSRCs have to be performed.

## Introduction

Birdshot Retinochoroiditis (BSRC) is a rare form of non-infectious posterior uveitis, in which recurrent inflammatory episodes affect retina and stromal choroid^1^. Patients with BSRC present with mainly bilateral typical hypopigmented choroidal ‘birdshot lesions’ around the fundus, which are suspected to be associated with the formation of lymphocytic aggregations (foci) in the choroid, optic nerve, and along the retinal vasculature^2^. Clinically, the patients suffer from floater, blurred vision, and sensitivity to light (photopsia). Over the many years of the course of the disease, the patients develop visual impairment including decreased visual acuity, peripheral vision, blindness at night (nyctalopsia) and blindness for colors (dyschromatopsia). Although this rare disease is clinically well characterized, the etiology and the pathophysiology of BSRC remain uncertain.

Evidences from human and mouse studies suggest that both, the innate and adaptive immune system contribute to the manifestation of non-infectious posterior uveitis, like BSRC^3–5^. Due to the strong association of BSRC to the human lymphocyte antigen (HLA) class I A*29.2 (relative risk of 224.35), a particular role in disease pathology can be attributed to T cells^6^. Indeed, analyses of vitreous fluids revealed the presence of retina and choroid-reactive intraocular CD4^+^ and CD8^+^ T cells in disease-active BSRC eyes proving their presence in an ongoing auto-inflammatory response^8^. Kuiper and colleagues further strengthened this body of evidence by the discovery of certain polymorphisms in the endoplasmic reticulum aminopeptidase (ERAP) gene of BSRC patients leading to imbalanced ERAP 1 and 2 function, thus influencing peptide-presentation of HLA-A*29 to T cells^7^. ERAP enzymes are necessary to process peptides for loading onto major histocompatibility complex I (MHC-I) on nucleated cells prior to surface transport. MHC-I presented peptides in turn are then recognized by CD8^+^ T cells that can initiate an immune response, thus generating immunological memory. Further supportive evidences were presented in studies showing improvement of ocular inflammation and visual acuity by corticosteroid-sparing immunomodulatory therapies^8–16^.

It was shown that the CD4^+^ T helper subset T_H_17 might be important to sustain chronic inflammation in various autoimmune diseases, such as psoriasis, inflammatory bowel disease, rheumatoid arthritis, and multiple sclerosis^17–20^. In line with these studies, T cells of BSRC patients produced IL-17 in response to human and choroid lysate and IL -17 secreting CD4^+^ and CD8^+^ T cells have been demonstrated to be enriched in the periphery of BSRC patients^21–23^. In addition, increased IL-17 and pro-inflammatory cytokines were presented in aqueous humor and serum of BSRC patients, suggesting IL-17 pathway activity^4,24^. However, most of these results were only noticeable in disease-active, treatment-naïve BSRC patients only. This may hint at a role rather in the establishment of auto-inflammatory episodes in BSRC pathology and excludes IL-17 producing T cells as marker for BSRC disease activity. Other cellular parameters have to be identified as markers for disease activity.

Several studies utilized distinct chemokine receptor expression patters to describe multiple subsets of CD4^+^ and CD8^+^ T cells^25,26^. Chemotactic receptors might give additional or more precise information about the abundance and origin of certain peripheral T cell compartments in BSRC disease. To gain insights in the T cell biology of BSRC, we conducted a study enrolling 11 BSRC patients that underwent clinical examination using multimodal imaging techniques and disease staging^27^. In parallel, we examined the abundance of certain memory T cell subsets. To assess relevant CD4^+^ and CD8^+^ T cell subsets, we analyzed the cell fractions directly ex vivo by multi-color staining of a set of chemokine receptors (CCR7, CXCR3, CCR4, CCR6 and CCR10)^26^. Comparison of immunological parameters to control donors reflected an altered composition of the T cell compartment of BSRC patients indicating that the disease could be monitored via phenotyping peripheral T cell populations. We furthermore correlated the resulting immunological phenotype with the clinical presentation of each BSRC patient on the sample collection day, intending to identify different immune signatures during clinical active or inactive state of disease.

## Methods

### Patients and study design

In our pilot study, we examined 11 patients (22 eyes) with BSRC. The diagnosis of BSRC was made according to the research criteria of the international consensus conference^28^. All patients with BSRC were previously tested for HLA-A29.2 allele positivity. Clinical examination was performed using multimodal standard imaging techniques, as previously published^27^. On the clinical examination day, visual acuity (VA), findings on slit lamp, indirect fundoscopy, spectral-domain optical coherence tomography (SD-OCT), fluorescein angiography (FA), and indocyanine green angiography (ICGA) were recorded. ICGA images were analyzed for the presence of hypofluorescent dots^27^. SD-OCT, FA, and ICGA were performed on the spectral-domain OCT (SPECTRALIS, Heidelberg Engineering, Heidelberg, Germany). Fundus photographs were taken with Zeiss FF 450 + . In parallel, 30 mL blood was collected from each BSRC patient and subjected to flow cytometric analysis of surface markers for inflammatory T cell subsets and for their effector function upon polyclonal stimulation. The study was performed in accordance with the Declaration of Helsinki and approved by the local ethics committee (Ethikkomission der Charité- Universitätsmedizin Berlin) (EA2/148/15). Written informed consent was obtained from each participating patient before blood sampling.

### Clinical parameters used for the study

The ocular inflammation was classified according to the Standardization of Uveitis Nomenclature (SUN) Working Group criteria^28,29^. Data of indirect fundoscopy were used to determine the degree of cell infiltration e.g. vitreous haze (VH) per eye. The degree of VH is classified as no infiltration = 0, very mild = 0.5, mild = 1, moderate = 2 and severe = 3. SD-OCT was used to determine deep resolution structural information, such as cystoid macular edema (CME) and central retinal thickness (CRT). CME was defined as the presence of intraretinal or subretinal fluid. FA and ICGA were simultaneously performed and analyzed by using proposed scoring system to classify the active intraocular inflammatory process by retinal, macular and optic disc leakage^30,31^. The degree of retinal leakage is classified as focal = 1 or diffuse = 2. The degree of macular leakage is classified as no perifoveal hyperfluorescence = 0, incomplete perifoveal hyperfluorescence = 1, mild 360° hyperfluorescence = 2, moderate 360° hyperfluorescence = 3, severe 360° hyperfluorescence with the hyperfluorescent area being approximately 1.5 disc diameter across = 4^30,31^. ICGA images were analyzed for the presence of hypofluorescent dots as sign for activity^32^. Four out of 11 patients were repeatedly (2-time points) analyzed in order to follow the course of the disease. The control cohort consisted of healthy mid-aged as well as elderly volunteers with Fuchs Endothelial Corneal Dystrophy (FECD) (n = 14).

### Blood sampling

Per study participant, 30 mL blood was collected in Li-heparin vacutainer tubes (BD, USA). Blood was diluted 1:1 with PBS/BSA and peripheral blood mononucleated cells (PBMCs) were separated by Bicoll-Paque (Biochrom, Germany) gradient centrifugation (20 min, 850xg at RT).

### PBMC culture

*In-vitro* polyclonal activation was performed with fresh PBMCs that rested overnight in culture medium, consisting of RPMI 1640 (Gibco, USA) supplemented with 10% heat inactivated human AB-serum (Lonza, Switzerland), 100U penicillin and 0.1 mg/ml streptomycin (Biochron, Germany). 5 × 10^6^ PBMCs/ml were either left unstimulated and served as control or were stimulated in culture medium supplemented with 10 ng/ml PMA and 1 µg/ml ionomycin (Sigma, Germany) for 6 h in an incubator (37 °C, humidified 5% CO_2_). After 2 h of stimulation, 2 µg/ml Brefeldin A (Sigma, Germany) was added to all samples.

### Flow cytometry

1 × 10^7^ PBMCs were labelled ex vivo on the cell surface with fluorescent monoclonal antibodies titrated to their optimal concentration: CD4-A700 (RPA-T4, BD, USA), CD8-V500 (RPA-T8, BD, USA) and CD45RA-PeCy7 (HI100, Biolegend, USA), CCR7-A488 (G043H7, Biolegend, USA), CCR4-PerCpCy5.5 (L291H4, Biolegend, USA), CCR6-BV605 (G03E3, Biolegend, USA), CCR10-PE (6588-5, Biolegend, USA), and CXCR3-A647 (G025H7, Biolegend, USA) for 15 min in the dark. For dead cell exclusion, 0.4 µM DAPI was added directly prior measurement. All stainings were performed in the presence of 1 mg/ml Beriglobin (CSL Behring, USA). All steps were performed at room temperature.

For the detection of cytokines, stimulated and unstimulated PBMCs were washed twice with PBS and dead cells were subsequently stained with FarRed (Molecular Probes, USA) for 20 min in the dark. Following surface staining antibodies were added during the last 10 min of the staining: Panel 1A: CD8-V500 (RPA-T8, BD, USA), CD4-PerCpCy5.5 (OKT 4, Biolegend, USA), CD45RO-BV785 (UCHL1, Biolegend), CCR7-A488 (G043H7, Biolegend, USA). Panel 2A: CD4-A700 (RPA-T4, BD, USA), CD8-V500 (RPA-T8, BD, USA), CD3-PE (UCHT 1, home), CD45RA-PeCy7 (HI100, Biolegend, USA), CCR7-A488 (G043H7, Biolegend, USA) followed by fixation and permeabilization with FACS-lysing/-perm2 solution (BD, USA) according to the manufacturer's protocol. Intracellular staining was conducted for 30 min in the dark with: Panel 1B: CD40L-APC (5C8, Miltenyi, Germany), IFN-γ-A700 (B27, BD, USA), IL-17-APCCy7 (BL168, Biolegend, USA), IL-4-PE (8D4-8, Biolegend, USA), IL-22-efl450 (22URTI, eBioscience, USA), IL-2-PeCy7 (MQ1-17H12, Biolegend, USA), CD3-efl605 (OKT3, eBioscience, USA). Panel 2B: TNF-α-PerCpCy5.5 (MAb11, Biolegend, USA), and CD69-PB (FN50, Biolegend, USA), CD40L-APC (5C8, Miltenyi, Germany). 2.5 × 10^6^ PBMCs were recorded on LSR II (BD, USA) flow cytometer, except unstimulated controls where 1 × 10^6^ PMCs were recorded. LSR II was equipped with 4 lasers and 15 fluorescent detection channels. Rainbow Calibration particles (6-peaks, BD, USA) were acquired daily, to ensure constant laser and measurement parameters. In brief, the high and low intensity bead populations for 5 different channels have to position into pre-defined gates and form sharp peaks, with a robust Coefficient of Variation, (rCV) below 5%. Acquired FACS data sets were analysed with FlowJo version 10 (Tree Star, USA).

### Statistical analysis

All data were analyzed using R or GraphPad Prism 7 (GraphPad Software, La Jolla, CA). We used Student’s t-test with Welch’s correction, Mann Whitney U Test, one-way ANOVA, and Spearman’s Rho correlation coefficient. A significant p-value was defined as p ≤ 0.05. Particular test usage is indicated in the figure legends.

## Results

### Patient characteristics

Eleven patients with BSRC were examined at the Department of Ophthalmology, Charité Berlin. All patients were Caucasians and showed HLA-A29.2 positivity (100%). Seven patients were female (64%, 7/11), and the mean age was 60 ± 9 years (range 39–73) on the examination day. All 11 patients were healthy at the day of blood collection, except for their ocular symptoms. The control cohort was initially selected to cover the typical age range of BSRC patients^1,5^, hence the control group consisted of healthy mid-aged volunteers and elderly subjects with FECD (n = 14, mean 60 years, range 31–87).

### Group classification

Based on previous studies, BSRC patients were classified in two groups: active and inactive disease^27^. We identified 10 eyes of 5 patients with retinal vascular leakage, hyperfluorescence of the disc in FA, and manifestation of dark dots in ICGA, which were summarized into the active intraocular inflammatory group. 12 eyes of 6 patients demonstrated no retinal vascular leakage in FA and dark dots in ICGA, and were thus graded as inactive intraocular inflammatory group. These eyes revealed an inactive, end-stage disease because of altered vascular architecture and retinal thinning^27^. Three eyes revealed a chronic CME, which last over years without any signs of vasculitis or choroiditis, so that these patients were graded as inactive. The patients of the inactive disease cohort have an average disease duration of 13 years compared to 4 years in patients with active disease. Most of the patients were on systemic immunosuppressive treatments: antimetabolites—mycophenolate mofetil (MMF): 4/11 (36%), calcineurin inhibitor—ciclosporine A (CsA): 1/11 (9%), prednisolone < 10 mg per day: 1/11 (9%), and MMF combined with prednisolone < 10 mg per day: 2/11 (18%). A total of 4 of 11 patients (36%) received adalimumab, a tumor necrosis factor-alpha inhibitor. More details about patients´ characteristics are presented in Table 1.

**Table 1:** Patients characteristic.

Patient	Age	Gender	Year of diagnosis	Disease status	Prior treatment	Current treatment	New switched treatment	Comorbidity
1	66	F	2006	Inactive	Corticosteroids	MFF	Adalimumab	
2	58	M	2003	Inactive	Corticosteroids	CSA	Adalimumab	Diabetes mellitus Typ II
3	59	M	2008	Active	Corticosteroids, CSA	MFF	Adalimumab	
4	65	F	2009	Inactive	Corticosteroids, CSA	MMF	Adalimumab	
5	63	F	1999	Inactive	Corticosteroids, CSA, MMF, adalimumab	−	−	
6	56	F	2015	Active	Corticosteroids, CSA	MMF	−	
7	72	F	1994	Inactive	Corticosteroids	MMF	−	
8	73	M	2015	Inactive	MFF	MMF	−	
9	59	F	2008	Active	Corticosteroids	−	−	
10	57	F	2016	Active	Corticosteroids	−	−	
11	39	M	2015	Active	Corticosteroids	CSA	−	Beta-Thalassemia

*CSA* ciclosporine A, *MFF* mycophenolate mofetil.

### Peripheral CD8^+^ T_EMRA_ T cell populations are enriched in patients with inactive BSRC disease

To achieve an efficient discrimination of peripheral naïve and memory T cell phenotypes and to delineate the composition of inflammatory subsets among CD4^+^ helper (T_H_) and cytotoxic CD8^+^ T cells (T_C_) of our BSRC cohort, we examined the expression of 6 different surface markers by flow cytometry (CCR7, CD45RA, CXCR3, CCR4, CCR6, CCR10) in freshly isolated peripheral T cells.

**Figure 1:**
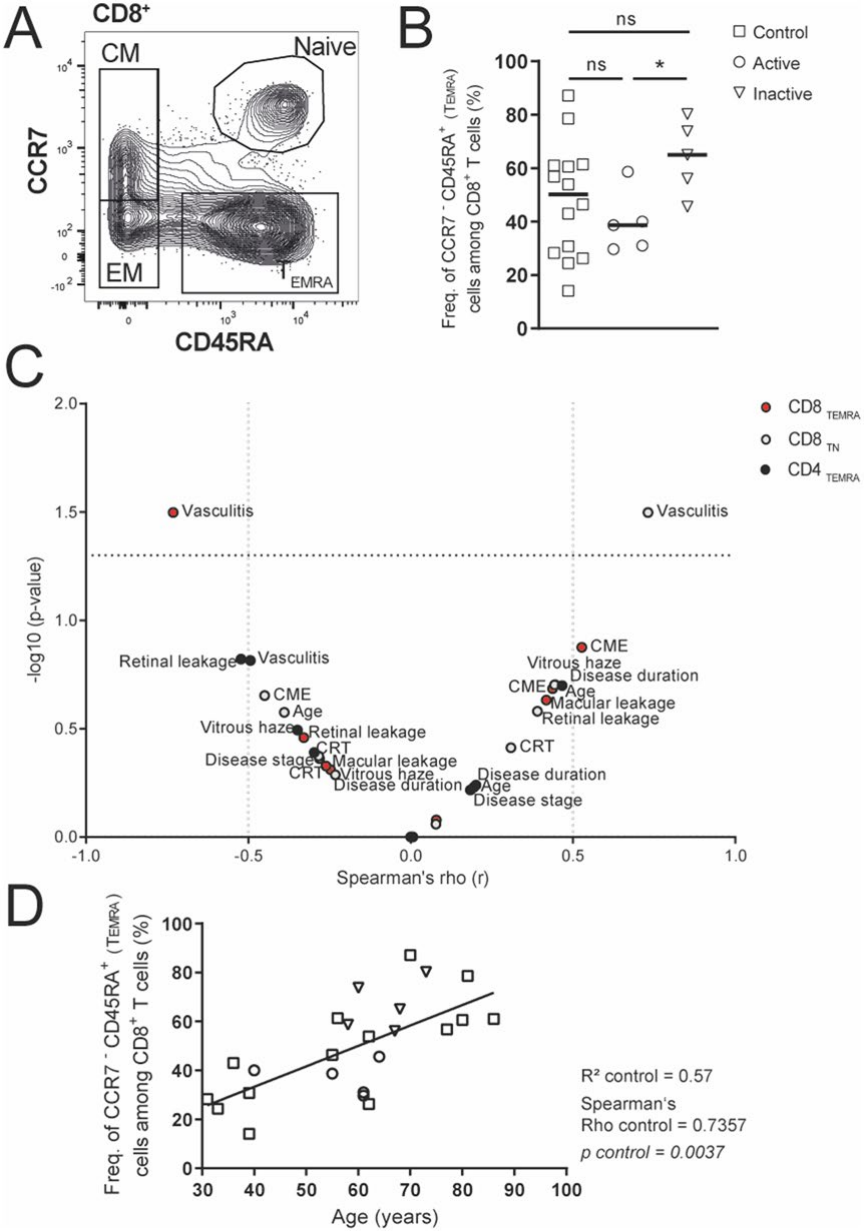
The activity state of BSRC affects peripheral CD8^+^ T_EMRA_ cells. (**A**) Exemplary strategy of gating CD8^+^ T-cell stages: T_CM_ (CCR7^+^CD45RA^−^), T_EM_ (CCR7^−^CD45RA^−^), naïve (CCR7^+^CD45RA^+^), and T_EMRA_ (CCR7^−^CD45RA^+^). (**B**) Blood analysis of peripheral CD8^+^ T_EMRA_ cells from controls compared to disease-active and -inactive BSRC patients. Lines indicate the median. Statistical analysis was performed using Student’s t-test with Welch’s correction (*p* < 0.05*). (**C**) Volcanoplot (− log10) shows Spearman’s Rho correlation coefficient of immunological *versus* clinical parameters of significant altered CD4^+^ and CD8^+^ T-cell populations in BSRC patients. (**D**) Linear regression of CD8^+^ T_EMRA_ cells with age of active and inactive BSRC patients and control probands. The population of CCD7−/CD45RA− was in one inactive patient absent, therefore the (**B**) and (**D**) include n = 5 inactive patients. *BSRC* birdshot retinochoroiditis, *CM* central memory, *CME* cystoid macula edema, *CRT* central retinal thickness, *EM* effector memory, *Freq.* frequency, *NS* not significant, *TEMRA* terminally differentiated effector memory CD45RA^+^ T cells.

The averaged proportions of CD4^+^ (55%) and CD8^+^ (21%) T cells among living lymphocytes were similar between the BSRC patient group and age-matched controls (49.6% and 19.8%; data not shown). According to the expression of CD45RA and CCR7, naïve, and memory T cell subsets were identified within the CD4^+^ and CD8^+^ T cell compartments, respectively: CCR7^+^CD45RA^+^ naïve T cells, CCR7^+^CD45RA^−^ central memory T cells (T_CM_), CCR7^−^CD45RA^−^ effector memory cells (T_EM_) and CCR7^−^CD45RA^+^ T_EMRA_ cells (gating strategy exemplified in Fig. 1A). Analysis of naïve vs. T_CM_, T_EM_, and T_EMRA_ memory T cell populations revealed significant differences in naïve vs*.* terminally differentiated T_EMRA_ CD8^+^ T cells within the BSRC group (Fig. 1B,C). Although median frequencies plus ranges of T_EMRA_ CD8^+^ T cells were similar between BSRC patients and the aged-matched control group (BSRC: median: 48% (30–80); control: median: 51.8% (14–87)), intragroup differences of the BSRC cohort were identified when disease activity was considered. Compared to the active disease group and treatment independent (*p* = *0.40*) (Supplementary Fig. S1), BSRC patients with inactive disease displayed an increased T_EMRA_ population in blood (median active: 39.6% to inactive: 64.1%, *p* = 0.01 *). The population of CCR7-/CD45RA- was absent in one inactive patient, therefore the Fig. 1B,D include n = 5 inactive patients. But this patient had CCR7 + /CDRA− cells, so further analysis could be conducted. Although not significant, we observed a tendency for an even higher median frequency of the CD8^+^ T_EMRA_ population in inactive eyes (median: 64.1%) compared to the control group (median: 51.8%) (Fig. 1B, p = *0.08*), suggesting a trend for accumulation of T_EMRA_ cells in disease-inactive episodes of BSRC disease. We observed a correlation of CD8 + T_EMRA_ frequencies with age in the control group (Spearman ‘s Rho = *0.73, p* = 0.003 **; Fig. 1D), but not in the BSRC group (Spearman’s Rho = *0.43, p* = *0.13 ns*; Fig. 1D). The BSRC patients belong to the same age group, and hence the differences in CD8 + T_EMRA_ frequencies can be attributed rather to disease state. 5/5 data points of the inactive group led profoundly over the R^2^ linear regression line, whereas 4/5 data points of the active group led under the R^2^ linear regression line, suggesting the possibility that CD8^+^ T_EMRA_ T cells indicate disease status of BSRC disease (Fig. 1D). Extended analysis of possible correlations of frequencies of CD8^+^ T_EMRA_ cells with clinical parameters of BSRC patients in a volcano plot showed a negative correlation with the presence of intraocular vasculitis (Spearman’s Rho = − *0.73 , p* = 0.03 *; Fig. 1C), but correlated only weakly negative and insignificantly with other active disease parameters, such as the presence of intraocular VH (Spearman’s Rho = − *0.25, p* = *0.49 ns*), CRT (Spearman’s Rho = − *0.30, p* = *0.47 ns*), retinal leakage (Spearman’s Rho = − *0.33, p* = *0.35 ns*), CME (Spearman’s Rho = *0.54, p* = *0.13 ns*), macular leakage (Spearman’s Rho = *0.42, p* = *0.23 ns*), optic disc leakage (Spearman’s Rho = *0.08, p* = *0.87 ns*), and disease duration (Spearman’s Rho = *0.44, p* = *0.20 ns*) (Fig. 1C). In contrast to the increased CD8^+^ T_EMRA_ cell population observed in inactive BSRC patients, active BSRC patients showed a significantly increased naïve CD8^+^ T cell population in blood (median 34.9%) compared to disease-inactive BSRC patients (11.8%, *p* = *0.01* *) and controls (22%, *p* = *0.03* *) (data not shown), that correlated significantly positive with vasculitis (Spearman’s Rho = *0.73, p* = 0.03*; Fig. 1C). Furthermore, we found weak positive correlations with clinical measures of active disease: VH (Spearman’s Rho = *0.44, p* = *0.20, ns*), retinal, and macular leakage (Spearman’s Rho = *0.39, p* = *0.26, ns*). Moreover, we noted a similar tendency for increased proportions of T_EMRA_ CD4^+^ T cells in inactive BSRC patients (median: 1.7%) compared to active BSRC patients (0.5%) and controls (0.9%) (data not shown) that was again independent from treatment and trended towards a similar correlation pattern as observed for CD8^+^ T_EMRA_ (Fig. 1C).

### Signs of an imbalanced T_H_1/T_H_2 subsets in patients with active eyes

**Figure 2:**
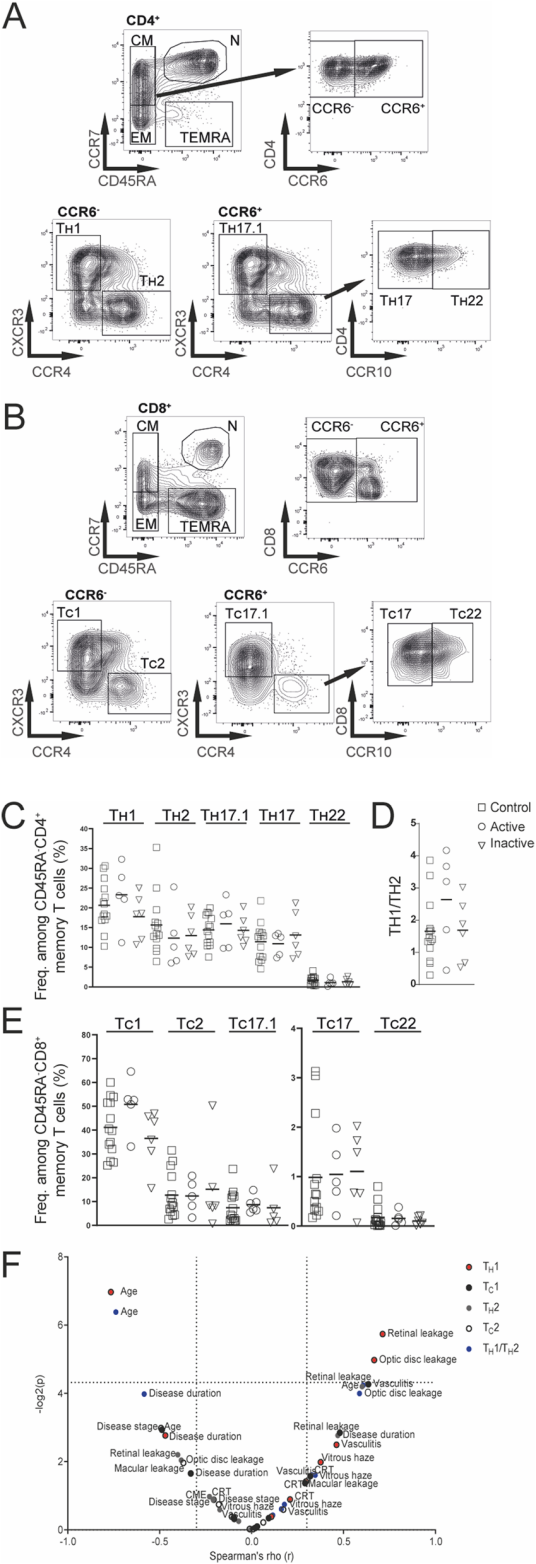
Inflammatory T-cell subsets in BSRC blood. (**A**) CD4^+^ and (**B**) CD8^+^ CD45RA^−^ central and effector memory T cells were used to gate CCR6^+^ and CCR6^−^ fractions. CCR6^−^ cells were further distinguished in T1, based on CXCR3 expression or T2, based on CCR4 expression. CCR6^+^ cells were distinguished in T17.1, based on CXCR3 expression and T17 and T22 subsets were gated based on CCR4 and CCR10 expression. (**C**) Lines show median frequencies of CD4^+^ T_H_1, T_H_2, T_H_17.1, T_H_17 and T_H_22 T-cell subsets of controls and active/inactive BSRC patients. (**D**) The frequencies of T_H_1 and T_H_2 fractions were used to calculate a T_H_1/T_H_2 ratio. (**E**) Lines show median frequencies of CD8^+^ T_C_1, T_C_2, T_C_17.1, T_C_17 and T_C_22 T-cell subsets. (**F**) Volcanoplot (− log10) shows Spearman’s Rho correlation coefficient of immunological *versus* clinical parameters of T_H_1, T_H_2, T_C_1, T_C_2 T-cell populations and T_H_1/T_H_2 ratio of BSRC patients. *BSRC* birdshot retinochoroiditis, *CM* central memory, *CME* cystoid macula edema, *CRT* central retinal thickness, *EM* effector memory, *N* naïve, *T*_*C*_ cytotoxic T cell, *T*_*H*_ T-helper, *TEMRA* terminally differentiated effector memory CD45RA^+^.

To assess the potential contribution of different CD4^+^ and CD8^+^ T cell subsets in BSRC pathogenesis, we further analyzed the circulating T cell memory compartment for inflammatory subsets. The combined CD45RA^−^ T_CM_ and T_EM_ memory T cell gates were further subdivided into the CD4^+^ helper and cytotoxic CD8^+^ T cell subsets T_H_1/T_C_1, T_H_2/T_C_2, T_H_17.1/T_C_17.1, T_H_17/T_C_17, T_H_22/T_C_22 using chemokine receptors: CXCR3, CCR4, CCR6, and CCR10 (Fig. 2A,B), as previously described^26^.

Within the BSRC patient group having active disease, we observed tendency for a predomination of CXCR3^+^ T cell subsets T_H_1/T_C_1, T_H_17.1/T_C_17.1 among both, CD4^+^ (median T_H_1: 23.1% and T_H_17.1: 18.2%; *p* = *ns*) and CD8^+^ T cell fractions (median T_C_1: 52.6% and T_C_17.1: 8%; *p* = *ns*) compared to the control group (median T_H_1: 19.7% and T_H_17.1: 14.65%; T_C_1: 40.1% and T_C_17.1: 4.1%) (Fig. 2C–E). In contrast, BSRC patients with inactive disease rather resembled the frequencies of inflammatory T cell subsets of control probands (median T_H_1: 18.5% and T_H_17.1: 13.4%; T_C_1: 39.7% and T_C_17.1: 3.36; *p* = *ns*), except a slightly increased frequency of T_C_17 cells (median 1.08%) compared to the control group (median 0.56%; *p* = *ns*). Of note, we found a slight, insignificant trend for an increased CCR6^+^ CD4^+^ (*p* = 0.39) and CD8^+^ T cells (*p* = 0.69), of which T_H/c_17, T_H/c_17.1 and T_H/c_22 subsets were gated (Supplementary Figs. S2) compared to the control group. Furthermore, we found tendencies for a skewed T_H_1/T_H_2 ratio in the active BSRC patient group, which was absent in the inactive BSRC patient group (Fig. 2D). Correlation of CXCR3^+^ inflammatory T cell populations T_H_1, T_C_1, and T_C_17.1 with clinical parameters revealed positive correlations in the volcano plot for these subsets with clinical parameters of an ongoing inflammatory episode in BSRC eyes, such as retinal leakage, optic disc leakage, and the degree of severity of the disease (Fig. 2F), although lacking significance.

### Analysis of T cell effector function

**Figure 3:**
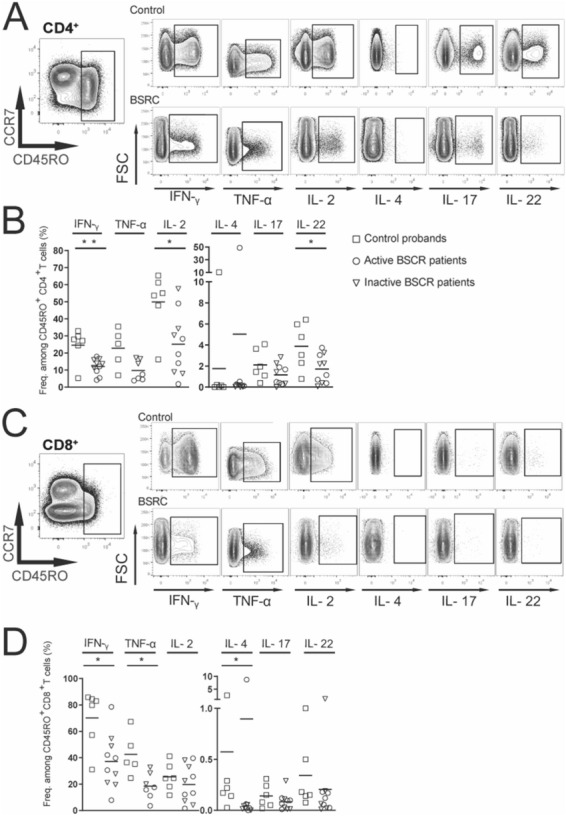
Reduced potential of BSRC memory T-cells to exert effector function. (**A**) Exemplary gating strategy of CD45RO^+^ memory T-cells and exemplary gating strategy of different cytokines after 6 h polyclonal activation in CD4^+^ (**A**) and CD8^+^ (**C**) T cells in control probands (upper row) and patients (lower row) respectively. (**B**,**D**) Analysis of IFN-γ, TNF-α, IL-2, IL-4, IL-17, and IL-22-producing CD45RO^+^ T-cell fractions in controls and BSCR patients. Lines indicate median. Mann Whitney U test was used for statistical analysis *p* < 0.05*. *BSRC* birdshot retinochoroiditis, *IFN-γ* interferon gamma, *IL* interleukin, *TNF-α* tumor necrosis factor alpha.

Next, we compared the overall effector function of peripheral memory T cells of BRC patients and the control group upon in vitro polyclonal stimulation using phorbol 12-myristate 13-acetate (PMA) and ionomycin. We assessed the capacity of the memory T cell pool to produce effector cytokines by intracellular staining of IFN-γ and TNF-α (mainly secreted cytokines of T_H_1/T_C_1 and T_H_17.1/T_C_17.1 polarized cells); IL-2, IL-4 (T_H_/T_C_2), IL-17 (T_H_17.1/T_C_17.1; T_H_17/T_C_17; T_H_22/T_C_22), and IL-22 (T_H_22/T_C_22) according to the preceding ex vivo characterization^26^. The evaluation of the intracellular staining of different cytokines derived from the CD45RO^+^ CD4^+^ and CD8^+^ memory T cell populations (Fig. 3A–D) indicated a reduced potential of BSRC patients to produce certain effector cytokines upon polyclonal stimulation compared to the control group (upper rows compared to lower), which was independent of disease activity (data not shown). Especially the levels of IFN-γ (*p* = 0.02*), IL-2 (*p* = 0.03*), and IL-22 (*p* = 0.04*) of CD45RO^+^ CD4^+^ memory T cells were significantly decreased (Fig. 3B). The amount of TNF-α reached a close to significant difference (*p* = *0.07*) between the patient cohort and the control probands. This trend continued in the CD45RO^+^ CD8^+^ T cell population of the BSRC patient group. Here, we observed also significantly lower IFN-γ (*p* = 0.007**), TNF-α (*p* = 0.01*), and IL-4 (*p* = 0.03*) levels, compared to the control group (Fig. 3D).

## Discussion

This pilot study represents a deep-phenotyping of various inflammatory memory T cell fractions in the peripheral blood of BSRC patients with active and inactive stage of disease and their comparison to control probands with an age range covering the typical age range of BSCR. We evaluated the composition of the circulating T cell compartment directly ex vivo and furthermore assessed the capacity of the memory T cell compartment to exert effector function upon polyclonal stimulation by measuring cytokine secretion profiles using flow cytometry.

First, we identified a slight increase of CD45RA-expressing CD8^+^ T cells (T_EMRA_) in blood of the inactive, end-stage group compared to the active group, although our sample size is too small to draw clear conclusions. Several studies characterized T_EMRA_ cells as low IL-2 producer and high IFN-γ and TNF-α secretor, accompanied by a high cytotoxicity and a high sensitivity to apoptosis^33–35^. Expansion of pathogenic T_EMRA_ cells have been proven in a variety of diseases from autoimmunity (multiple sclerosis, lupus) to allotransplantation and bone regeneration^36–39^. Yap et al. reported that expansion of T_EMRA_ CD8 can be detected in kidney-transplant recipients despite a long-term stable graft function and that such expansion is associated with a two-fold higher risk of kidney graft dysfunction^40^. Accumulation of CD8^+^ T_EMRA_ cells is also associated with age, chronic antigen stimulation, and immune system senescence, which is often interpreted as a hallmark of aging and a marker of exhausted immune system^41–45^. Our data did not show any correlation between CD8^+^ T_EMRA_ T cell abundance and age, but the frequencies of CD8^+^ T_EMRA_ cells correlated negatively with the presence of vasculitis *e.g.* the extent of intraocular inflammation. It can be assumed that a high amount of T cells with CD8^+^ T_EMRA_ phenotype describe an impaired immune function in the eye likely as a consequence of the recurrent and constant inflammatory episodes which are described in BSRC. This raises the question of whether CD8^+^ T_EMRA_ is associated with a higher risk of worse manifestation of BSRC or it could be an indicator for a poor prognostic outcome due to already a rarefication of the vessels and thinning of the retina. Reinke et al. demonstrated that delayed fracture healing significantly correlated with enhanced levels of CD8 T_EMRA_ in peripheral blood^39^. These cells seem to be directly involved in the pathogenesis in poor healing^44^. CD8^+^ T_EMRA_ might be exhausted, senescent, and poorly proliferative T cells that display several functional abnormalities.

Thus, CD8^+^ T_EMRA_ cells could be probably proposed as a biomarker that describes an advanced BSRC disease state. Further prospective studies are necessary to investigate whether CD8^+^ T_EMRA_ T cells occur only in inactive, burned-out patients or it is also found in newly diagnosed patients as a potential risk factor.

Furthermore, the clear HLA association in BSRC implies a pivotal role of T cells in disease development and/or progression^6^. Histopathological descriptions characterized lymphocytic aggregations with their foci in the deep choroid, in the optic nerve head, and along the retinal vasculature^2^. In addition, analyses of eye infiltrates derived from vitreous fluidics revealed the presence of retina or choroid-reactive intraocular CD4^+^ and CD8^+^ T cells in disease-active BSRC eyes, suggesting their role in ongoing auto-inflammatory processes^8,21^. Expanded T cell clones showed effector memory phenotype and were able to secrete the classical T_H_1-cytokine profile (IL-2, IFN-γ and TNF-α) upon CD3 stimulation^8^. The leukocytes include mostly ocular specific T cells that synergistically contribute to local inflammation and tissue damage^46,47^. Usually, the exposure of the highly immunogenic ocular antigens (for example retinal S-antigen) to T cells is prevented by the retina-blood barrier and the immune privileged environment of the eye. When these barriers breach, ocular specific T cells may migrate into the eye and induce tissue damages. Different reports demonstrated intraocular T cell reactivity to retinal and choroidal lysate, but the antigen in BSRC is currently still unknown^8^. However, most of the studies classify the T cells by distinct cytokines^3,21,22^. Due to reoccurring episodes and probably exhausted T cells, it is questionable whether measuring the effector function by cytokine profile analysis is a suitable approach to classify the T cell function of uveitis patients. Here, the chemotactic receptors might give additional or even more precise information about the abundance and the composition of certain peripheral T cell compartments in BSRC disease. In general, chemotactic gradients can be established elsewhere in the body by many different cell types in consequence to immunological activation. The expression of a certain set of chemokine receptors makes T cells restricted to specific chemokines, thus ensuring correct guidance and compartmentalization of antigen-primed T cells as demonstrated by Loyal et al.^26^. Our results show a trend for the predomination of the CXCR3^+^ T cell subsets T_H_1/T_C_1 in both, CD4^+^ and CD8^+^ T cell fraction. The T1/T2 imbalance indicate changes in the immune function and has been described in experimental autoimmune uveitis/uveoretitinitis (EAU)^48^. Therefore, T1/T2 ratio may serve as an indicative marker for BSRC disease activity. Of note, most of the studies measure the concentration of cytokine as secreted by T_H_1 and T_H_2 cells while we demonstrate a possible contribution of CD8^+^ T cells as well as gradually impaired cytokine secretion highlighting the importance of an ex vivo cell classification. Moreover, recent studies revealed a prominent role of T_H_17 cells that drive chronic inflammation in T cell-associated immune disorders including non-infectious uveitis and BSRC^22,49^. In BSRC a subset of CD8^+^ T cells was reported that express the endothelial adhesion molecule ´MCAM` (CD146) and secrete IL-17^23^. This suggest that T_H_17 and T_C_17 cells may concomitantly contribute to the IL-17 mediated pathogenesis. It would be of interest to evaluate IL-17 production of intraocular T cells in BSRC, since retinal IL-17 production CD8^+^ T cells in EAU has been demonstrated by Peng et al.^50^ Tc17 cells might be able to target HLA-molecules that present ERAP2 trimming-dependent epitopes, linking the HLA I presentation with IL-17 associated immunity in BSRC^7^. Our data did not reveal a clear tendency for T_H_17/T_C_17 phenotypes. However, the current role of IL-17 in BSRC is not clarified, but few studies showed that IL-17 has protective effects during inflammation^51–53^.

Finally, we observed impaired cytokine production of CD4^+^ and CD8^+^ memory T cells in BSRC patients regardless of their treatment and stage of disease. These results support in the literature reported elevated levels of IL-23, IL1- β, IL-6, and TGF- β in serum of BSRC patients which might be associated with the T cell exhaustion^24,25^. Molins et al. demonstrated elevated levels of IL-17A in patients with BSRC in remission with and without immunomodulatory treatment. Patients who were not receiving immunomodulatory treatment had significantly higher levels of circulating IL-23 and TGF-β1 than patients under immunomodulatory treatment or healthy subjects^54^.

Of course, many studies reported about immune perturbations in the peripheral blood of uveitis patients and the immune changes in the peripheral blood not necessarily mirror the changes in the eye. But immune privilege is not interpreted as a lack of immunosurveillance^55–57^. It is known that immune cells visit healthy sites of immune privilege^58,59^.

## Conclusion

To sum up, we observed more CD8^+^ T_EMRA_ in inactive BSRC patients and significantly less in active patients. Therefore, high frequencies of CD8^+^ T_EMRA_ T cells could be an indicator for a poor prognostic outcome in advanced stage of the disease. The T1/T2 imbalance in BSRC may indicate autoimmune processes and decreased cytokine levels in the periphery are probably caused by immunosuppression or exhaustion of the memory T cell subsets.

The size of the herein analyzed patient pool is too small for definitve conclusions. Therefore, immunological studies with higher patient numbers are required for a deeper understanding of the T cell contribution to BSRC development and the potential for targeted immunotherapy. However, our results offer new insights into the immunological pathophysiology of BSRC disease and may help in defining new biomarkers for monitoring for this potentially blinding disease. Accordingly, we propose to distinguish these cells ex vivo based on the expression of chemokine receptor instead of functional analyses.

## Supplementary Information

Supplementary Figure 1.

Supplementary Figure 2.

Supplementary Information 3.
