# Supplementary figures and images for: Analysis of peripheral inflammatory T cell subsets and their effector function in patients with Birdshot Retinochoroiditis
Source: Sci Rep. 2021 Apr 21;11:8604. doi: 10.1038/s41598-021-88013-0 (PMC8060342; doi:10.1038/s41598-021-88013-0)

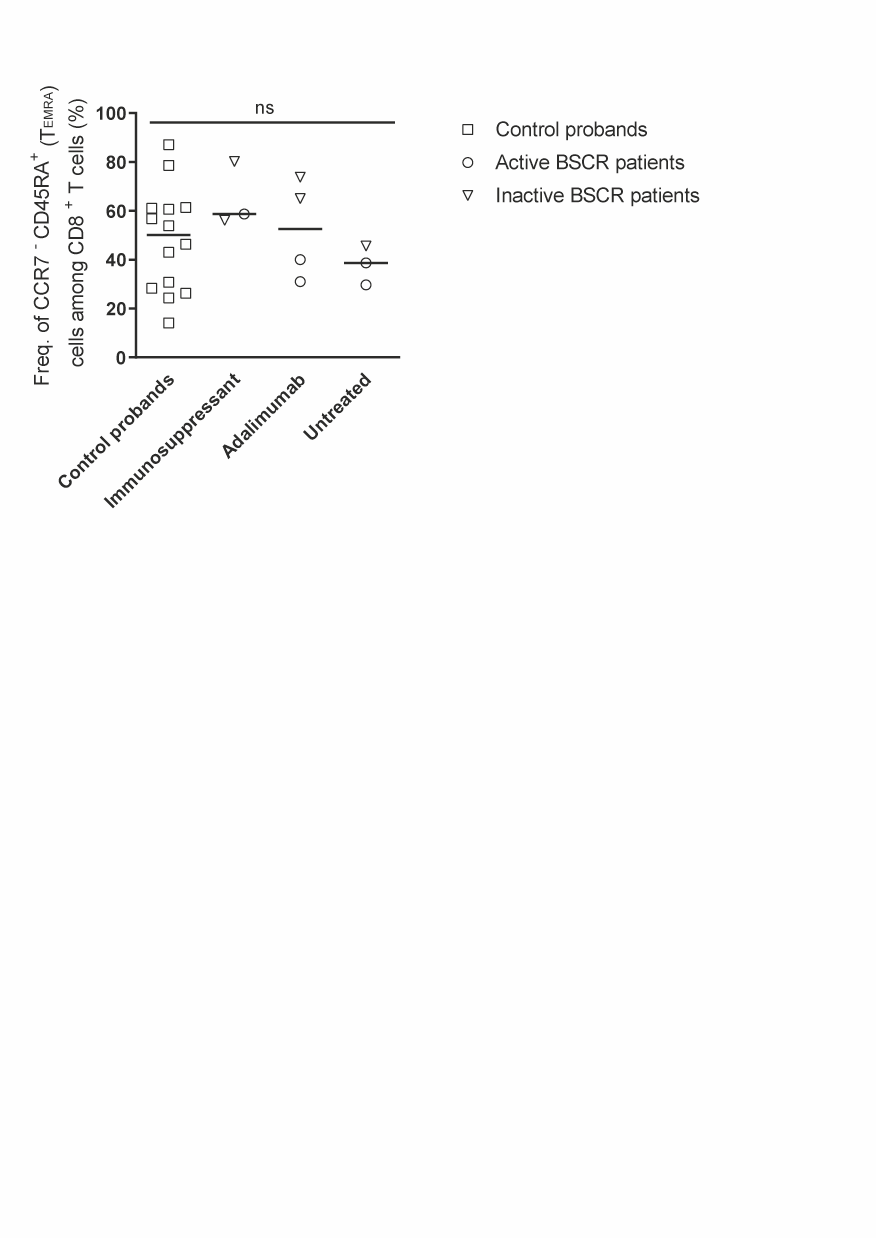

Supplement: Supplementary file 1 — Supplementary Figure 1. [file 41598_2021_88013_MOESM1_ESM.tiff]

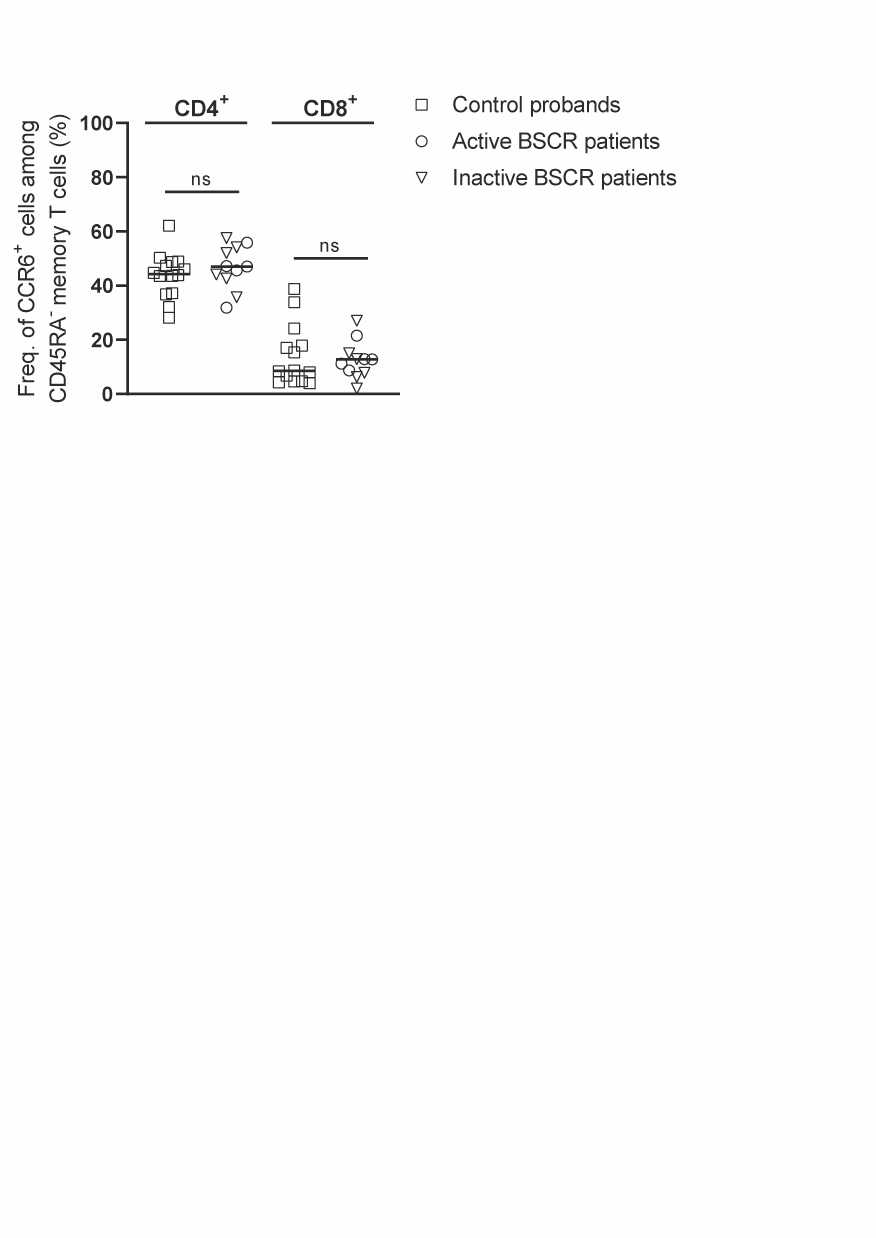

Supplement: Supplementary file 2 — Supplementary Figure 2. [file 41598_2021_88013_MOESM2_ESM.tiff]
