# Supplementary material for: Analysis of peripheral inflammatory T cell subsets and their effector function in patients with Birdshot Retinochoroiditis
Source: Sci Rep. 2021 Apr 21;11:8604. doi: 10.1038/s41598-021-88013-0 (PMC8060342; doi:10.1038/s41598-021-88013-0)
Supplement: Supplementary file 3 — Supplementary Information 3. [file 41598_2021_88013_MOESM3_ESM.docx]

**Supplementary Figure 1. T_EMRA_ CD8^+^ T cells according to treatment of individual BSCR patients of the study.** Frequencies of CCR7^-^CD45RA^+^ T_EMRA_ CD8^+^ T cells were grouped according to treatment status of individual BSCR patients (Table 1) of the study. One-way ANOVA test was used for statistical analysis (*p*<0.05).

BSRC, birdshot retinochoroiditis; TEMRA, terminally differentiated effector memory CD45RA^+^

**Supplementary Figure 2. CCR6-expression on CD4^+^ and CD8^+^ T cells which comprise the T_H/C_17, T_H/C_17.1 and the T_H/C_22 subsets.** CCR6^+^ T cells gated from combined CM and EM T cell gates. Student’s t-test with Welch’s correction was used for statistical analysis (*p*<0.05).

CM, central memory; EM, effector memory; T_C_, cytotoxic T cell; T_H_, T-helper
